# Oxidative Stress and Antioxidants in Chronic Rhinosinusitis with Nasal Polyps

**DOI:** 10.3390/antiox12010195

**Published:** 2023-01-14

**Authors:** Junhu Tai, Jae-Min Shin, Jaehyung Park, Munsoo Han, Tae Hoon Kim

**Affiliations:** 1 Department of Otorhinolaryngology—Head & Neck Surgery, College of Medicine, Korea University, Seoul 02841, Republic of Korea; 2 Mucosal Immunology Institute, College of Medicine, Korea University, Seoul 02841, Republic of Korea

**Keywords:** oxidative stress, antioxidants, chronic rhinosinusitis, mucosa, research progress

## Abstract

Oxidative stress results from an imbalance between the production of reactive oxygen species and the body’s antioxidant defense system. It plays an important role in the regulation of the immune response and can be a pathogenic factor in various diseases. Chronic rhinosinusitis (CRS) is a complex and heterogeneous disease with various phenotypes and endotypes. Recently, an increasing number of studies have proposed that oxidative stress (caused by both environmental and intrinsic stimuli) plays an important role in the pathogenesis and persistence of CRS. This has attracted the attention of several researchers. The relationship between the presence of reactive oxygen species composed of free radicals and nasal polyp pathology is a key topic receiving attention. This article reviews the role of oxidative stress in respiratory diseases, particularly CRS, and introduces potential therapeutic antioxidants that may offer targeted treatment for CRS.

## 1. Introduction

Oxidative stress can be defined as an imbalance between the production and degradation of reactive oxygen species (ROS) [1]. In the process of generating energy through aerobic respiration, humans and other mammals produce a variety of ROS [2], such as hydrogen peroxide and superoxide anions. Excess ROS can be eliminated through the action of various enzymes, including that of superoxide dismutase (SOD), catalase, and other components, to be maintained within the normal range. However, under oxidative stress, excessive ROS levels cause cell damage through interactions with proteins, lipids, and nucleic acids, thereby negatively affecting the function and structure of tissues [3]. Various physiological and pathological conditions have been related to oxidative stress; the failure of the antioxidant system and an increased production of ROS have been linked with obesity, aging, and some mucosal diseases [4]. Recently, the impact of oxidative stress on the human mucosal system has received increasing attention from researchers, especially with regard to respiratory mucosa. Studies have shown that the house dust mite can induce ROS production while inhibiting antioxidant responses in bronchial epithelial cells [5]. In asthma cases, ROS play a key role in the persistence and amplification of airway inflammation and promote mucus hypersecretion, increased vascular permeability, and airway remodeling [6]. In addition to their impact on the lower airway, ROS also have a variety of effects on the nasal mucosa of the upper airway. The nasal submucosal gland is a source of multiple molecules that are important for mucosal host defense [7]. Oxidative stress may play a crucial role in causing the dysfunction or impairment of the nasal epithelial barrier [8]. Chronic sinusitis (CRS) is a disease that stems from a variety of pathogenic factors. Among its common causes are environmental stimuli (such as pollution); viral, fungal, or bacterial infection; smoking; or physiological factors, such as genetic predisposition and immunodeficiency [9]. Patients with CRSwNP suffer from a variety of distressing symptoms, including nasal congestion, decreased or lost sense of smell, rhinorrhea, posterior rhinorrhea, and facial pressure or pain [10]. As a result of the shared type 2 inflammatory pathway, patients with CRSwNP often have comorbid asthma and/or nonsteroidal anti-inflammatory drug-exacerbated respiratory disease, which lead to the need for repeated treatment with corticosteroids and/or sinonasal surgeries to alleviate their uncontrolled symptoms [11,12]. However, the treatment often fails to achieve satisfactory results, which means that it is urgent to find new causes and treatments for CRSwNP. The treatment options of CRSwNP include medical or surgical therapy, and biological agents have been approved or subject to recent clinical trials, but no antioxidant has been approved to treat CRSwNP yet [13]. The antioxidant capacity of the human respiratory mucosa plays an important role in the etiology of CRS, especially CRS with nasal polyps (CRSwNP) [14]. Key factors in the innate defense mechanism of the upper respiratory tract include SOD, peroxiredoxin-2 [15], bactericidal/permeability-increasing fold-containing family A member 1 (BPIFA1) [16], and adenylate-cyclase-activating polypeptide receptor 1 (ADCYAP1) [17,18].

With the implications of ROS being known to the public, people have very high expectations for the provision of antioxidants that can prevent ROS generation. Antioxidant-rich nutrients are generally used as supplements to reduce the damage caused by ROS [19]. Recent studies regarding the role of antioxidants in CRS, especially CRSwNP, have confirmed acceptable results in this regard. For example, a study involving 32 patients with CRS established that local treatment with antioxidants had a better effect on accelerating the recovery of patients’ nasal sinus mucosa after surgery than other conventional topical treatments [20]. This review discusses the role of oxidative stress in respiratory diseases, especially CRS. We also highlight potential antioxidants that may be used to treat CRS as indicated by the latest research.

The study searched PubMed, Web of Science, and Scopus, using a combination of the following search terms (in Title/Abstract): “chronic rhinosinusitis”, “oxidative stress”, and “antioxidant”. This review did not limit the types of research included. Basic research and clinical research are both included. The inclusion criteria include articles exploring the relationship between CRSwNP, oxidative stress, and antioxidants through basic or clinical research published in English between 1992 and 2022. Moreover, there are no restrictions on the types of articles. Article types, such as review, original research, letters, communications, and editorials, are all included in this review, and only some articles for which full text was unavailable were excluded. All selected articles have been imported into EndNote, which will intelligently delete duplicate articles.

## 2. Oxidative Stress in Respiratory Diseases

The respiratory system is vulnerable to oxidative stress owing to its complex conductive airways and large alveolar surface area [21,22]. Airway oxidative stress is widely defined as an imbalance between the pro-oxidant and antioxidant processes in the airways [23]. The main pathogenic factors causing diseases comprise infection and inflammation, protease and antiprotease imbalances, and oxidative stress overwhelming the body’s antioxidant defenses [24]. Increased lung oxidative stress in respiratory diseases may occur due to heightened levels of exogenous or endogenous oxidants, or due to a reduction in endogenous antioxidants (Table 1). Exogenous oxidants include air pollution, tobacco smoke, biomass smoke, allergens, particulate matter, and fine particulate matter (<10 μ, <2.5 μ, <0.1 μ in diameter) [25,26]. Endogenous oxidants include hydrogen peroxide, peroxynitrite, xanthine oxidase, superoxide anions, mitochondrial oxidants, and myeloperoxidase [27,28]. Antioxidants that may experience reduced levels in the body include thioredoxin, nuclear factor erythroid 2-related factor 2 (Nrf2), glutathione, forkhead box protein, vitamins, and SOD [29,30]. Oxidative stress drives respiratory diseases through several mechanisms, including activation of the proinflammatory transcription factor nuclear factor-κB (NF-κB) pathway, generation of autoantibodies to carbonylated proteins, reduced expression of sirtuin-1, DNA damage, reduced activity of antiproteases, and increased release of transforming growth factor-beta [31].

The respiratory tract is a direct window of contact with the external environment. Pollutants or toxic substances, such as nitrogen dioxide, sulfur dioxide, and particulate matter in the air, may cause asthma symptoms [32], and these substances are more or less related to oxidative stress. There is evidence that oxidative stress is a prevalent factor in asthma [33,34]. An antioxidative–prooxidative imbalance may lead to pathological changes in the respiratory epithelial cells, and other disease states (such as airway hyperresponsiveness) and defects in the intracellular antioxidant defense system may contribute to asthma development [35]. Methods for detecting oxidative stress-related biomarkers in asthma comprise tests of the exhaled condensate, bronchoalveolar fluid, systemic circulation, and urine as well as experimental detection (Table 2).

Exhaled breath condensates and bronchial fluid detection are airway-related methods for detecting several biomarkers related to oxidative stress [36,37]. It was found that the concentrations of hydrogen ions, hydrogen peroxide, nitric oxide, and 8-isoprostane in the breath of patients with asthma were generally higher than those in healthy controls, and these results were easily detected in patients with severe asthma [38]. A review of 46 studies also revealed that changes in the concentrations of hydrogen ions, hydrogen peroxide, and nitric oxide in exhaled air were related to the deterioration of allergic asthma in children [39]. The level of glutathione determines whether the T helper type-1 (Th1) or type-2 (Th2) immune response mode is dominant, with glutathione depletion being conducive to Th2-related reactions [40]. Acute asthma attacks lead to a decrease in the glutathione levels in children’s exhaled breath condensates, and these levels increase after steroid treatment [41]. A study that analyzed the role of oxidants in human lung injury found that the baseline levels of 3-bromotyrosine in bronchoalveolar fluid proteins from individuals with mild allergic asthma were slightly higher than those in a control group; after exposure to the segmental allergen challenge, the 3-bromotyrosine content in the bronchoalveolar fluid of these individuals increased by more than 10-fold [42]. Catalase enzyme is another key antioxidant. Its activity in the bronchoalveolar lavage fluid of asthmatic patients is lower than that in healthy controls, with a similar observation having been made in animal experiments [43].

One study evaluated the various components of enzymatic and non-enzymatic antioxidants using ELISA to evaluate the levels of glutathione peroxidase and SOD enzymes in patients’ blood and using high-performance liquid chromatography to measure reductions in the levels of glutathione [44]; the glutathione peroxidase and SOD levels were significantly low in children with asthma. Another study involving 57 asthmatic and 38 healthy participants investigated asthma-related markers via noninvasive methods. Urinary levels of bromotyrosine and F2-isoprostane increased in asthmatic patients, indicating that these compounds may be associated with asthma [45]. As for experimental detection, researchers found that a redox thiol/dithiol imbalance alters copper-zinc SOD levels in the cells of asthmatic patients and that copper-zinc SOD was easily inactivated by hydrogen peroxide [46]. In a mouse model of asthma, copper-zinc SOD transgenic mice exhibited less airway inflammation and hyper-reactivity [47].

The inhalation of pollutants increases the oxidative load in individuals with asthma, and oxidative damage of the airway epithelium along with activation of innate immune mechanisms lead to allergic sensitization and inflammation. In addition, the intracellular redox imbalance causes a disruption of signaling cascades and cellular responses, thus increasing the airway inflammation and promoting airway remodeling and hyperresponsiveness [48]. Researchers have used N-acetylcysteine to remove excess ROS and promote endogenous antioxidant mechanisms. It successfully reduced airway inflammation and hyperresponsiveness in animal models of asthma, similarly reducing airway hyperresponsiveness in healthy human subjects and patients exposed to diesel exhaust particles [49,50]. In other studies, however, antioxidant treatments had no effect on asthmatic symptoms. Sulforaphane plays a key role in preventing oxidative stress and inflammation [51]. A double-blind, randomized trial involving 40 adults compared the effects of sulforaphane on airway inflammation and oxidative stress [52]. In contrast to what was expected, the sulforaphane intake did not improve any clinical features of pulmonary inflammation, oxidative stress biomarkers, or asthma atopy. Studying the interaction between currently available asthma and antioxidant treatments (focusing, for example, on the interaction between corticosteroids and ROS) may help in developing novel therapeutic interventions. Corticosteroid treatment is related to a reduction in airway oxidative stress in patients with asthma [53]. However, long-term corticosteroid treatment can lead to mitochondrial dysfunction, which leads to ROS-mediated cardiovascular, metabolic, and other complications [54,55]. Although the treatment of oxidative stress has great potential in assisting with asthma therapy, only a few studies have achieved success in this regard, with most research failing to uncover obvious benefits.

We previously reviewed the effects of oxidative stress and antioxidants on allergic rhinitis, which, similar to asthma, is regarded as a united airways disease. We introduced transcription factors, such as Nrf2 and NF-κB, in a mouse model of allergic rhinitis and in nasal mucosa epithelial cells of patients with allergic rhinitis. Several possible therapeutic antioxidants, such as sulforaphane, resveratrol, and taurine, were also tested, with the latter showing promising results through inhibiting oxidative stress markers [56]. In the following section, we will introduce the role of oxidative stress and antioxidants in CRS, another united airway disease [57].

## 3. CRS and Oxidative Stress

### 3.1. Endotype and Phenotype of CRS

**Figure 1:**
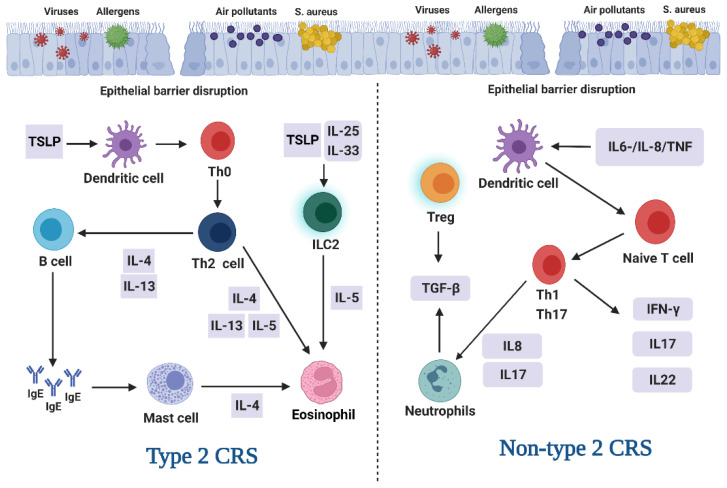
The type 2 and non-type 2 endotypes of CRS.

CRS can be classified into type 2 and non-type 2 CRS, based on the differences in immune responses between Th1/Th17 and Th2 cells [58]. Previous studies have concluded that CRS without nasal polyps (CRSsNP) shows neutrophilic predominance, whereas CRSwNP exhibits eosinophilic predominance [59]. However, contrary to these findings for European and American patients, most Asian patients with CRSwNP possessed neutrophil predominance, and CRSsNP showed an immune response favoring type 2 CRS and type 1 or 3 inflammation [60]. As illustrated in Figure 1, type 2 CRS is characterized by epithelial cell disorder and elevated levels of Th2 cells (which produce interleukin (IL)-4, IL-5, and IL-13 cytokines), B cells, dendritic cells, and eosinophils [61,62]. The nasal sinus epithelium is the main source of thymic stromal lymphopoietin, which plays a key role in type 2 inflammation by activating Th2 cells and group 2 innate lymphoid cells [63]. IL-5 promotes eosinophil inflammation, whereas IL-4 and IL-13 activate homotypic transformation and mucus production in CRSwNP [61]; B cells produce IgE and other immunoglobulins [62]. It has also been reported that the levels of type 2 inflammatory cytokines IL-25 and IL-33 originating from other epithelial sources are increased in type 2 CRS [64]. Unlike type 2 CRS, non-type 2 CRS is associated with a significant increase in Th1/Th17 cells, corresponding with elevated levels of interferon (IFN)-γ, IL-8, and IL-17 cytokines [65]. IL-6, IL-8, and tumor necrosis factor stimulate the production of IFN- γ and further IL-8 by T cells to strengthen the immune response; IL-8 recruits neutrophils into this region, which release more cytokines, and the epithelial response to environmental stimuli leads to the activation of dendritic cells, thereby inducing the differentiation of Th1 and Th17 cells [66]. According to its phenotype, CRS is currently classified as either expressing or lacking nasal polyps (NPs) [67]. Of the two, CRSwNP is usually accompanied by a dysfunction of the nasal mucosal fibers responsible for mucus transportation and self-cleaning, which leads to the inability of ciliated cells to lubricate the epithelium and remove impurities [68]. This change in the innate defense mechanism of the upper respiratory tract relates to the etiology of CRSwNP, with the antibacterial and antioxidant capacity of the tract showing a gradual decline as CRSwNP progresses [69]. Along with the decline in antioxidant capacity, oxidative stress plays a crucial role in the pathogenesis of CRSwNP [70,71].

### 3.2. Oxidative Stress in Chronic Sinusitis with NPs

**Figure 2:**
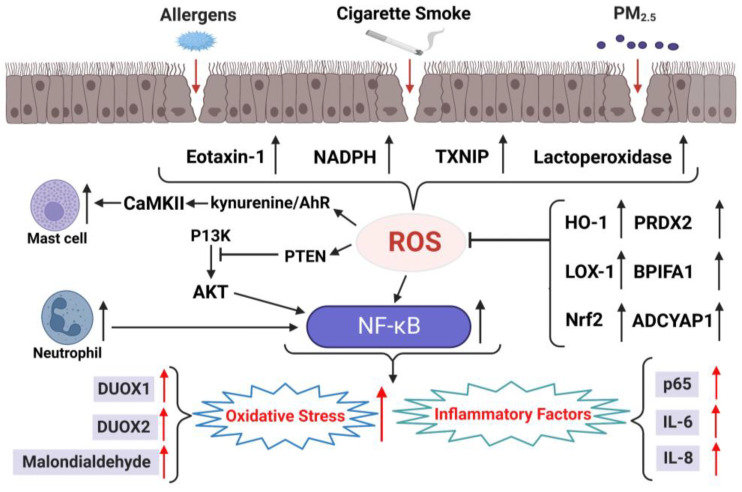
Role of oxidative stress in CRSwNP.

Exogenous factors that induce oxidative stress in CRS (as they do in other respiratory diseases) include tobacco smoke, allergens, and fine particulate matter [72,73]. These exogenous oxidants can reduce the permeability of airway epithelial cells and destroy the nasal sinus epithelial barrier [74], ultimately inducing oxidative stress (Figure 2). Inflammatory chemokines play a key role in coordinating inflammation [75] and are regulated by redox reactions [76]. Eotaxin-1, a member of the CC chemokine family, induces eosinophil recruitment and activation. One study observed eotaxin immunoreactivity in epithelial and endothelial cells of NPs [77]. Another study showed that eotaxin-1 plays a key role in the selective recruitment of eosinophils in NPs [78]. Nicotinamide adenine dinucleotide phosphate (NADPH) oxidase produces ROS that are involved in oxidative stress and signal transduction [79]. A recent study evaluated the expression of the NADPH oxidase subunit p67^phox^ and the oxidative stress marker 4-hydroxy-2-nonenal (4-HNE) in NP tissues of 13 patients with CRSwNP and the nasal mucosae of nine healthy controls. The expression levels of both were significantly higher in NP tissues than those in healthy mucosae [80], confirming that lipid peroxidation occurred in NP tissues. Thioredoxin-interacting protein (TXNIP) is a multifunctional protein that can also increase ROS production and induce oxidative stress by inhibiting the activity of thioredoxin, an antioxidant [81]. One study confirmed that TXNIP expression is upregulated in patients with CRSwNP, indicating the protein’s key role in the pathogenesis of CRSwNP [82]. Heme oxygenase (HO)-1 has been proposed to be a cytoprotective enzyme against oxidative stress in CRSwNP [23]. In a study involving 40 patients with CRSwNP and 20 healthy controls, the expression levels of HO-1 mRNA and proteins were significantly higher in the NPs of the patients than those in the nasal mucosae of controls [83]. Scavenger receptors recognize various wastes and foreign materials invading the human body and usually counteract the generation of ROS induced by environmental toxins [84]. Lectin-like oxidized LDL receptor-1 (LOX-1) is one such scavenger receptor, and its expression is induced by oxidative stress [85]. LOX-1 mRNA expression was significantly higher in patients with CRSwNP than that in healthy controls of one study [86], emphasizing its crucial role in the redox regulation of CRSwNP.

ROS cause neutrophil activation, which can upregulate the expression of genes to release their corresponding proteins. Many of these genes depend on the activation of transcription factors, such as NF-κB, to induce expression. NF-κB activation may be the basis for the effect of pro-inflammatory stimulation of human neutrophil gene expression [87]. The growth of NPs is also closely related to the effects of various cytokines [88], and NF-κB is one of the most important factors related to the production of cytokines during inflammation. One study analyzed the expression of NF-κB, associated inflammatory cytokines, and adhesion molecules in patients with CRSwNP [89]. NPs of a CRSwNP group possessed a significantly higher number of NF-κB p65-positive cells and higher mRNA expression levels of IL-6, IL-8, and eotaxin than uncinate tissues of the control and CRSsNP groups. Nrf2 acts as the key regulator of oxidation and environmental stress by translocating to the cell nucleus and promoting the expression of genes that produce a cell protective response [90]. Nasal sinus mucosal barrier function and tight junctions that had been destroyed by particulate matter could be restored via Nrf2 administration, indicating that activation of the Nrf2 pathway may be a potential therapeutic target for CRS [91]. Another study also showed that the barrier dysfunction of nasal sinus epithelial cells induced by cigarette smoke can be reversed through Nrf2 activation [92].

In addition to the above oxidative stress reactions related to CRSwNP, the following have been noted. Ca2+/calmodulin-dependent protein kinase II (CaMKII) is a multi-polymer serine-threonine kinase [93]. The normal activation of CaMKII triggers the exchange of subunits between these holoenzymes, but the increased activation of CaMKII via ROS [94,95] may lead to inflammation and other diseases. The kynurenine/AhR axis mediates mast cell activation through oxidative CaMKII in the pathogenesis of CRSwNP [96]. More in-depth research is needed to clarify the correlation between the kynurenine/AhR axis and CRSwNP-related oxidative stress.

The mucosal surface is protected by many antibacterial factors, such as lactoperoxidase, which creates an inhibitory action on bacteria through the production of ROS [97]. Lactoperoxidase requires H_2_O_2_ to oxidize thiocyanate, thus producing hypothiocyanite [98]. Another key process in epithelial defense is the regulation of dual oxygenase (DUOX) expression and function [99]. A study exploring the correlation between DUOX1 expression and inflammatory mediators in CRS [100] found that DUOX1 mRNA levels were significantly increased in patients with CRSwNP compared to those in healthy individuals or patients with CRSsNP. In fact, both the CRSwNP and CRSsNP groups exhibited higher DUOX2 mRNA levels than the control group. The H_2_O_2_ content was significantly high in patients with CRSwNP, with H_2_O_2_ levels in their nasal secretions being closely related to the expression level of DUOX. These nasal secretions similarly exhibited relatively high levels of cytokines, such as eotaxin, tumor necrosis factor-α, and IL-8. This study demonstrated that ROS generate an overexpression of DUOX1 and DUOX2 in patients with CRSwNP, and the authors postulated that DUOX1 and DUOX2 are key factors in innate defense signal transduction and nasal mucosal inflammation in the human nasal airway epithelium.

Edema is an important histological process in the pathogenesis of CRSwNP. Activated inflammatory cells and their secreted mediators cause tissue inflammation and edema and the waste of this chronic inflammatory process comprises free radicals that cause oxidative stress [101]. However, their exact role and impact on CRSwNP progression remain unclear. A study involving 24 patients with CRSwNP and 20 healthy controls investigated the impact of oxidative status on the severity of CRSwNP and associated quality of life parameters [102]. Oxidative stress level (measured as the total antioxidant status and nitric level) was significantly related to the degree of nasal congestion and disease severity. Another study compared the concentrations of malondialdehyde, SOD, and nitric oxide in healthy and NP tissue samples [70]. Compared to the control tissues, NP samples contained a significantly higher level of malondialdehyde and lower levels of SOD and nitric oxide. Malondialdehyde is the main end product of lipid peroxidation [103], while SOD and nitric oxide are key antioxidants [104]. This demonstrated a close correlation between oxidative stress and the pathogenesis of NPs.

In terms of antioxidant enzymes, the genetic expression of peroxiredoxin-2 (PRDX2), BPIFA1, and ADCYAP1 is crucial to the innate defense mechanism of the upper respiratory tract, in addition to that of SOD [69]. In a study performed in 2006, the levels of ADCAP1, BPIFA1, and SOD proteins were found to be differentially expressed in NPs of Chinese patients with CRSwNP and refractory CRSwNP, suggesting that the reduced expression levels of their genes may be related to the pathogenesis of CRSwNP [105]. In addition, a recent study reported that ADCYAP1, BPIFA1, and PRDX2 were differentially expressed in the nasal mucosa of Caucasian patients with CRSwNP [69], confirming that the differential expression of these genes reduced the antioxidant capacity in patients with CRSwNP. And it has been reported that PRDX2 is related to the severity of asthma, which is the one of the main clinical problems of CRSwNP [106]. Although the same study found that surgery and long-term local corticosteroid treatment can reverse the expression of the above target genes, these procedures can only partially alleviate the decline in the antioxidant capacity of the nasal mucosa, and there are still shortcomings in re-establishing normal SOD homeostasis. Moreover, several surgeries also represent one of the main clinical problems faced by patients with CRSwNP, and surgical treatment should not be a routine choice. It is necessary to determine the specific mechanisms underlying transcriptional and steroid-induced changes that affect the antioxidant capacity of nasal mucosa. There is evidence that the phosphatase and tensin homolog gene (PTEN) can inhibit the activation of phosphoinositide 3-kinase (PI3K), which in turn affects the phosphorylation of protein kinase B (Akt) [107]. The PI3K/PTEN/Akt signaling pathway regulates cell growth, apoptosis, proliferation, and metabolism [108] and is associated with various chronic inflammatory and autoimmune diseases [109]. An in vitro study investigated the role of PTEN in nasal epithelial cells under oxidative stress and the correlation between PTEN and the PI3K/Akt pathway [110]. Mouse nasal epithelial cells were treated with H_2_O_2_ to induce oxidative stress and create a cell damage model. In the cells injured by H_2_O_2_, oxidative stress was induced due to an increase in ROS levels and corresponding apoptosis, and, notably, this damage was aggravated by PTEN. The studies of oxidative stress and antioxidants related to CRSwNP are summarized in Table 3, and the biomarkers of oxidative stress and antioxidants related to CRSwNP are summarized in Table 4.

### 3.3. Therapeutic Antioxidants in Chronic Rhinosinusitis with NPs

Oxidative stress plays an important role in the pathogenesis of NPs. High levels of free radical-mediated lipid peroxidation metabolites have been observed in and is related to the severity of NPs [112,113]. Based on the impact of oxidative stress in the occurrence and development of CRSwNP, researchers have explored the feasibility of various antioxidants in the treatment of this disease, including flavones, resveratrol, and terpenoids (Table 5).

Flavones have proven antioxidant and anti-inflammatory effects [119,120]. Hariri et al. [110] found that several flavones such as apigenin inhibit the upregulation of Muc5AC and inducible nitric oxide synthase while also inhibiting the release of cytokines, such as IL-8. These effects resulted in an increase in the ciliary beating and mucociliary clearance of airway cells, supporting the clinical potential of flavones as therapeutic options for CRSwNP. Another candidate is resveratrol, which is a natural product extracted from a Peruvian legume plant that strongly inhibits cyclooxygenase, contributes to cancer prevention, and provides cardiovascular protection, among other beneficial effects [121,122]. Kim et al. used an eosinophilic CRSwNP mouse model to test the therapeutic effect of resveratrol, comparing it to that of triamcinolone acetonide; resveratrol significantly reduced eosinophil infiltration and the degree of subepithelial fibrosis in the nasal mucosa of mice, similar to the effect of triamcinolone acetonide [111]. Moreover, the expression levels of IL-4, IL-5, prostaglandin D synthase, and leukotriene C4 synthase were significantly reduced by resveratrol treatment, and high doses of resveratrol strongly inhibited the production of 5-lipoxygenase. These results demonstrate that resveratrol can prevent and treat eosinophilic CRSwNP through its antioxidant and anti-inflammatory effects. Nitric oxide is released in the nose and sinuses and is related to upper respiratory tract diseases. In allergic rhinitis, CRSsNP, and CRSwNP, the concentration of nitric oxide changes and is considered an indicator of disease severity [123]. Endothelial nitric oxide synthase (eNOS) plays an important role in vascular permeability, edema, and inflammation, and CRSwNP has been shown to increase eNOS phosphorylation [116]. 1,8-Cineol is a natural monoterpene with anti-inflammatory and antioxidant properties [124]. Researchers have found that 1,8-cineol significantly affects eNOS phosphorylation (and thereby, its subsequent activation), indicating that terpenoid antioxidation may have an effect on the treatment of CRSwNP. Quercetin is another bioactive compound with strong antioxidant activity that has been extensively studied [125]. It significantly increases trans-epithelial Cl^−^ transport and ciliary beat frequency in mouse and human nasal airway cells, demonstrating the feasibility of using quercetin for local administration to nasal sinuses [117]. Another treatment option is erdosteine, a drug that has already been approved for the treatment of acute and chronic lung diseases and was originally developed as a mucus-dissolving agent. It has antioxidant, anti-inflammatory, and antibacterial properties and can prevent or reduce the lung tissue damage caused by oxidative stress by regulating ROS production [126]. One study evaluated the efficacy of erdosteine in the treatment of CRSwNP and found that both the endoscopic results and questionnaire survey values of patients with CRSwNP improved after erdosteine treatment, relative to those of a control group. This confirms the feasibility of using erdosteine as an effective substitute for current drugs [118].

## 4. Conclusions

We reviewed the literature describing the effect of oxidative stress on CRSwNP and summarized several promising antioxidants for the treatment of CRSwNP. However, the current use of antioxidants in patients with CRSwNP is still limited, and there is a lack of evidence. In addition, there are obstacles and limitations in terms of research into antioxidant treatments, such as the small number of clinical cases and short research time. Now, there are many antioxidants for researchers to assess efficacy. The key is that this needs to be carried out in a multi-center, multi-population, and multi-stage study. In a word, further in-depth research is required to prove and support the development of antioxidants for CRS treatment.

## Figures and Tables

**Table 1:** Sources of oxidative stress in respiratory diseases.

Exogenous Oxidants	Endogenous Oxidants	Reduced Antioxidants
Air pollution	Hydrogen peroxide	Thioredoxin
Tobacco smoke	Peroxynitrite	Nrf2
Biomass smoke	Xanthine oxidase	Glutathione
Allergens	Superoxide anions	Forkhead box protein
Particulate matter	Mitochondrial oxidants	Vitamins
PM_2.5_ (<10 μ, <2.5 μ, <0.1 μ)	Myeloperoxidase	SOD

**Table 2:** Biological targets and biomarkers of oxidative stress in asthma.

Targets	Biomarkers
Exhaled breath condensate	Hydrogen ions
	Hydrogen peroxide
	Nitric oxide
	Oxides of nitrogen
	8-Isoprostanes
	Glutathione
Bronchoalveolar fluid	3-Bromotyrosine
	Catalase
Systemic circulation	Reduced glutathione
	Ascorbic acid
	α-Tocopherol
	Lycopene
	β-Carotene
Urine	Bromotyrosine
	F2-isoprostane
Experimental detection	Copper-zinc SOD
	Manganese SOD

**Table 3:** Summary of studies of oxidative stress and antioxidants related to CRSwNP.

Research Type	Key Findings of Basic Research Study and General Information of Clinical Trial	Reference
Basic research studies	Eotaxin immunoreactivity in endothelial cells of NPs	Yao et al. [77]
	Eotaxin-1 plays a key role in the selective recruitment of eosinophils in NPs	Yoshifuku et al. [78]
	Expression of p67phox and 4-HNE were higher in NP tissues than healthy mucosae	Zheng et al. [80]
	TXNIP expression is upregulated in CRSwNP	Lin et al. [82]
	Expression of HO-1 mRNA and proteins was higher in the NPs than that in control	Yu et al. [83]
	LOX-1 mRNA expression was higher in CRSwNP than that in healthy controls	Nishida et al. [86]
	CRSwNP group possessed a higher number of NF-κB p65-positive cells and higher mRNA expression levels of IL-6, IL-8, and eotaxin than control group	Jung et al. [89]
	The barrier dysfunction of nasal sinus epithelial cells can be reversed through Nrf2 activation	Tharakan et al. [92]
	DUOX1 mRNA levels were increased in CRSwNP compared to those in control	Cho et al. [100]
	Oxidative stress level was related to the nasal congestion and disease severity	Topal et al. [102]
	Compared to the control tis-sues, NP samples contained a higher level of malondialde-hyde and lower levels of SOD and nitric oxide	Cekin et al. [70]
Clinical trial	25 Caucasian patients (10 females and 15 males, aged 51–62 years). Moderate to high preoperative Malm endoscopy and Lund–Mackay CT scores. The treatment lasted for 6 months	Mihalj et al. [69]

**Table 4:** Biomarkers of oxidative stress and antioxidants in the CRSwNP.

Biomarkers of Oxidative Stress	Biomarkers of Antioxidants
Eotaxin-1 [77,78]	HO-1 [23,83]
NADPH [79,80]	LOX-1 [85,86]
TXNIP [81,82]	Nrf2 [90,91,92]
NF-κB [87,88,89]	SOD [70,102,104]
Lactoperoxidase [97,98]	PRDX2 [69,111]
DUOX1, DUOX2 [99,100]	BPIFA1 [69,111]
Malondialdehyde [70,102,103]	ADCYAP1 [69,111] PTEN, PI3K, Akt [107,108,109,110]

**Table 5:** Potential antioxidants for use in the treatment of CRS.

Antioxidants	Roles in the Nasal Mucosa and NPs	Reference
Several representative flavones (apigenin, wogonin, chrysin, tangeritin)	Inhibits the upregulation of Muc5AC and inducible nitric oxide synthase, as well as the release of cytokines (e.g., IL-8)	Hariri et al. [114]
Resveratrol	Decreases the degree of eosinophilic infiltration and subepithelial fibrosis, as well as levels of IL-4, IL-5, prostaglandin D synthase, and leukotriene C4 synthase	Kim et al. [115]
Terpenoids	Monoterpene oxide 1,8-cineol decreases the excessive eNOS phosphorylation typically found in NPs	Koennecke et al. [116]
Quercetin	Increases transepithelial Cl^−^ transport and ciliary beat frequency in culture models of sinonasal epithelium	Zhang et al. [117]
Erdosteine	This study compared patients with CRSwNP treated with Erdosteine alone or Erdosteine in combination with nasal corticosteroid spray and found that the response was significantly better in the Erdosteine-only group	Hoza et al. [118]
